# Assessing the relationship between reproductive autonomy and contraceptive use in rural Malawi

**DOI:** 10.1186/s12978-023-01688-8

**Published:** 2023-09-22

**Authors:** Alexandra Wollum, Marta Bornstein, Gladson Mopiwa, Alison Norris, Jessica D. Gipson

**Affiliations:** 1 Department of Community Health Sciences, Fielding School of Public Health, University of California Los Angeles (UCLA), UCLA Bixby Center on Population and Reproductive Health, Los Angeles, USA; 2 Division of Epidemiology, College of Public Health, The Ohio State University, Columbus, OH USA; 3 Department of Health Promotion, Education, and Behavior, Arnold School of Public Health, University of South Carolina, Columbia, USA; 4 Adolescent Girls and Young Women Program-The Global Fund Grant ActionAid Malawi, Lilongwe City, Malawi

## Abstract

Reproductive autonomy, or the extent to which people control matters related to their own sexual and reproductive decisions, may help explain why some people who do not intend to become pregnant nevertheless do not use contraception. Using cross-sectional survey data from 695 women aged 16 to 47 enrolled in the Umoyo Wa Thanzi (UTHA) study in Malawi in 2019, we conducted confirmatory factor analysis, descriptive analyses, and multivariable logistic regression to assess the *freedom from coercion* and *communication* subscales of the Reproductive Autonomy Scale and to examine relationships between these components of reproductive autonomy and current contraceptive use. The *freedom from coercion* and *communication* subscales were valid within this population of partnered women; results from a correlated two-factor confirmatory factor analysis model resulted in good model fit. Women with higher scores on the *freedom from coercion* subscale had greater odds of current contraceptive use (aOR 1.13, 95% CI: 1.03–1.23) after adjustment for pregnancy intentions, relationship type, parity, education, employment for wages, and household wealth. Scores on the *communication* subscale were predictive of contraceptive use in some, but not all, models. These findings demonstrate the utility of the Reproductive Autonomy Scale in more holistically understanding contractive use and non-use in a lower-income setting, yet also highlight the need to further explore the multidimensionality of women’s reproductive autonomy and its effects on achieving desired fertility.

## Introduction

Globally, 85 million pregnancies are unintended (i.e., mistimed or unwanted) every year, representing 40% of all pregnancies [1]. Unintended pregnancies are associated with a range of negative health and social impacts on women and their families [2]. Despite recent increases in the use of contraception, an estimated 26% of women in low- and middle-income countries who would like to prevent pregnancy are not using modern contraception [3]. In sub-Saharan Africa, approximately a third of all pregnancies are estimated to be unintended and approximately 25% of women who want to avoid pregnancy are not using modern contraception [4–6]. In Malawi, the site of this study, modern contraceptive prevalence among married women has increased drastically from 2000 to 2016 from 26 to 58%. Nevertheless 41% of pregnancies are unintended and couples, on average, have more children than they desire [7].

Gender dynamics and power within sexual relationships are important to understanding reproductive behavior and decision-making, including contraceptive use. Partner objection and partner disapproval have been documented as significant barriers to contraceptive use [8–11]. Intimate partner violence-including sexual violence (i.e., sexual abuse) has been linked to higher levels of unintended pregnancy [12–16]. There is now a large and growing body of research examining spousal communication about family planning, showing consistent and positive relationships with current contraceptive use [17–19]. These dynamics are particularly important to examine in Malawi. While past studies have shown individual level factors associated with higher odds of contraceptive use (i.e., urban residence, currently or formerly married, employed, higher education, perceptions of no or positive side effects [20–22]), multiple studies from Malawi have demonstrated that partner approval of contraception use and communication with a partner about family planning influence contraceptive use [23]. Qualitative studies further describe how partner support, gender dynamics within relationships, and perceived negative consequences of contraceptive use on sexual pleasure influence contraceptive decision making [8, 24, 25]. Moreover, preferences of the male partner can dominate reproductive discussions and decision-making [8].

Reproductive autonomy, defined as “the power to decide about and control matters associated with contraceptive use, pregnancy, and childbearing,” captures these dynamics [26]. In 2014, Upadhyay et al. developed a scale to measure reproductive autonomy. The Reproductive Autonomy Scale, developed and validated in the United States among women at contraceptive and abortion facilities, uses 14 items to measure three domains of reproductive autonomy: decision making, communication, and freedom from coercion. Decision making represents having the “primary say” in matters related to contraceptive use, pregnancy, and childbearing. Communication is defined as “feeling comfortable talking with one’s partners regarding contraceptive use, pregnancy, and childbearing” and freedom from coercion is the “absence of pressure from a partner in regards to contraceptive use, pregnancy, and childbearing” [26]. Each of these constructs is represented by a subscale. Several studies have fielded the Reproductive Autonomy Scale in part or in full in a low- or middle-income country [27–29]; however, the findings documenting the relationship between reproductive autonomy and contraceptive use are mixed. One study among Ghanian young women showed that decision-making was positively associated with contraceptive use at last sex [28] while another study in Vietnam found no relationship between any of the subscales and contraceptive use at last sex [29].

In this study, we examine reproductive autonomy among partnered women (ages 16–47 years) in a rural community in Central Malawi. In this context, fertility is often experienced within marriage. With a total fertility rate of 4.4 children per woman, half of women have given birth by age 19 [7]. Marriage, both monogamous and polygamous, is nearly ubiquitous and divorce and remarriage are common [30, 31]. The median age of marriage for women is 18.2 [7]. This paper has two aims: (1) to determine the reliability and construct validity of the Reproductive Autonomy Scale and two of its subscales in this population and (2) to determine whether reproductive autonomy was associated with contraceptive use among women in partnerships. Identifying if and how reproductive autonomy, as operationalized in this scale, is related to contraceptive use in a low-resource setting can provide insight into strategies to support women in choosing if and when they become pregnant, using a contraceptive method when desired, and aligning fertility preferences with outcomes.

## Methods

### Data

The data from this study come from the Umoyo wa Thanzi (UTHA) [Health for Life] research program, a longitudinal cohort study focused on sexual and reproductive health. The cohort was recruited from villages within a non-profit hospital’s catchment area in rural part of Lilongwe District (approximately 20,000 residents) in 2014. Eleven village clusters (19 villages) were selected by strata (rural, plantation, trading centre) for inclusion in the study. Every woman between the ages of 15–39 years living in the selected villages were invited to participate in the study [32]. Since 2014, four additional waves of surveys have been implemented with intermittent new recruitment. Additional information on the study methodology is explained in detail elsewhere [33]. We analyze data from the fifth wave, conducted with women in May–September 2019. All participants in the fifth wave had participated in at least one previous wave of data collection. The content of the Wave 5 survey was informed by previous quantitative findings from Waves 1–4 and by qualitative findings conducted in the same region in 2018 [34]. The survey focused specifically on issues related to perceptions of pregnancy risk, infertility, reproductive autonomy, and reproductive history.

The surveys were developed in English and translated into Chichewa through an iterative process with both English-speaking and bilingual English-Chichewa team members. Translations were reviewed for meaning and to ensure that items would be understood in the Malawian context, with the final wording determined through collaborative consensus [35]. Trained Malawian research assistants who spoke Chichewa conducted tablet-based surveys with participants at the participants’ home or in another private space chosen by the participant. Surveys took approximately 30 min to complete and participants were compensated with MK 2000, or approximately $2.00. This study was approved by the Institutional Review Boards (IRB) at The Ohio State University and Malawi College of Medicine.

### Analytic sample

Since the Reproductive Autonomy Scale is focused on decision making and behavior within the context of a partnership, the scale was only administered to women who had ever had sex. Given the scale’s emphasis on reproductive autonomy in the context of partnerships and our focus on contraceptive use, we excluded women who said they were single (n = 111), currently pregnant (n = 70), or who reported they had reached menopause (defined in the survey as no longer menstruating) (n = 4). Women over age 49 years (n = 9) were also excluded for a total analytic sample of 695. We also excluded respondents who reported they were sterilized in a sensitivity analysis.

### Measures

#### Reproductive autonomy

The Reproductive Autonomy Scale is comprised of three subscales: (1) *freedom from coercion*, (2) *decision making*, and (3) *communication*. Based on the input of the local UTHA research team, only the *freedom from coercion* and *communication* subscales were fielded in full in the UTHA Wave 5 survey [26] (Appendix Table 4). The *decision-making* subscale included questions about abortion and adoption decision-making, which local partners decided not to include given abortion was illegal in Malawi and adoption uncommon in the community. For the *freedom from coercion* subscale (5 items, e.g., My partner has pressured me to become pregnant) and *communication* subscale (5 items, e.g., It is easy to talk about sex with my partner), possible answers for the items within each subscale ranged from strongly disagree to strongly agree on a 4-point Likert scale and responses were assigned a score from 1 (lowest autonomy) to 4 (highest autonomy). Subscale scores were created by summing the items within each subscale. Higher numbers represented higher levels of reproductive autonomy, requiring the *freedom from coercion* subscale to be reverse coded.

#### Contraceptive use

Contraceptive use was assessed by asking the respondent “Currently, are you using any method to avoid pregnancy in your relationship?”.

#### Covariates

We used the question “All things considered, do you intend to become pregnant in the next 12 months?” as our primary measure of pregnancy intention. Responses included yes, no, and undecided. In sensitivity analyses, we also considered a measure of pregnancy happiness based on the question “How happy would you be if you became pregnant in the next year?”. Options included very happy, somewhat happy, and not at all happy.

Covariates also included number of living children (continuous), respondent’s highest level of education (continuous), and employment for wages in the past three months (yes/no) (informal employment, including agricultural work, and/or any formal employment), and relationship type (currently in monogamous marriage, currently in a polygamous marriage, or in a non-cohabitating relationship/engaged). We measured respondent household wealth by conducting a principal components analysis on measures of asset ownership (e.g., bicycle, mattress) and retained the first component based on plotting eigenvalues on a scree plot [36]. For purposes of describing the sample, we categorized this measure into quintiles.

### Analysis

#### Construct validity

To assess the construct validity of the Reproductive Autonomy Scale as a whole, we first examined the correlation matrix of all the items of the Reproductive Autonomy Scale included in the instrument. We then performed a confirmatory factor analysis to assess the factor structure. Using Lavaan software in R [37], we tested a two-factor model with both correlated and uncorrelated factors using the robust diagonally weighted least squares estimator [38]. Latent factors were standardized, allowing free estimation of all factor loadings. To assess model fit, we examined the confirmatory factor index (CFI), Tucker Lewis Index (TLI), root mean square error of approximation (RMSEA), and the standardized root mean square residual (SRMR) [39]. We tested model fit using chi-square goodness of fit tests. We also calculated Cronbach’s alpha as a measure of internal consistency of each of the subscales.

We then performed descriptive analyses to understand how these subdomains of reproductive autonomy were associated with sociodemographic characteristics, measures of future pregnancy intention, and contraceptive use and method type. We tested bivariate associations using chi-square tests of independence for binary variables and bivariable linear regressions for reproductive autonomy subscales.

#### Association between reproductive autonomy and contraceptive use

We constructed multivariable logistic regression models to test our hypothesis that reproductive autonomy and contraceptive use would be positively related. We controlled for factors known to influence contraceptive use, including pregnancy intentions, relationship type, parity, education, employment for wages, and household wealth [11, 20].

We tested an additional hypothesis that pregnancy intention moderated the relationship between reproductive autonomy and contraceptive use by constructing a model that included interactions between each reproductive autonomy subscale and pregnancy intention. We used likelihood ratio tests to compare models with interactions to main effects models without interactions.

To control for sampling at the village level, we clustered standard errors at the village-level. We present the unadjusted model including only the reproductive autonomy subscales, the model controlling for sociodemographic and partnership factors, and the fully specified model after testing for the salience of including an interaction with pregnancy intention. Model calibration was tested using a Hosmer–Lemeshow test.

Analysis was conducted in Stata 15 SE and R.

## Results

### Characteristics of the sample

The vast majority of respondents had children (> 96%) with the plurality of the sample having four or more children (34%) (Table 1). The mean age was 29.4 with respondent ages ranging from 16 to 47 years. Seventy six percent of respondents were married/currently living as married and reported that their husband only had one wife. Another 18% were in polygamous marriages, and 6% were in a non-cohabitating relationship or engaged.

**Table 1:** Sample characteristics, reproductive autonomy subscales, and contraceptive use by demographic characteristic

	N (%)	Mean freedom from coercion	p-value	Mean communication	p-value	% using contraceptive method (%)	p-value
Full sample	695 (100%)	16.8		17.7		91	
Living children							
No children	26 (3.8%)	15.5	Ref.	16.3	Ref.	65	Ref.
1 child	137 (19.8%)	17.1	< 0.01	17.6	0.02	92	0.003
2 children	146 (21.1%)	17.0	0.01	17.8	< 0.01	90	0.001
3 children	150 (21.6%)	16.6	0.09	17.7	0.04	92	0.001
4 + children	234 (33.8%)	16.8	0.01	17.8	0.01	94	0.003
Age (n = 529)							
< 20	27 (4.1%)	16.7	Ref.	17.3	Ref.	85	Ref.
20–24	145 (22.1%)	17.0	0.67	17.7	0.48	90	0.42
25–29	172 (26.2%)	16.7	0.96	17.5	0.65	91	0.25
30–34	126 (19.2%)	16.7	1.0	17.5	0.67	89	0.59
35 +	186 (28.4%)	16.8	0.86	18.0	0.21	94	0.13
Marital status							
Married with one partner	529 (76.1%)	16.9	Ref.	17.8	Ref.	92	Ref.
Married and partner has another wife	127 (18.3%)	16.7	0.50	17.5	0.29	92	0.94
In a non-cohabitating relationship/engaged	39 (5.6%)	15.6	< 0.01	16.9	0.01	79	0.03
Education							
No education	65 (9.4%)	15.7	Ref.	17.5	Ref.	89	Ref.
Less than standard	471 (67.8%)	16.9	0.02	17.8	0.20	91	0.50
Complete standard	67 (9.6%)	16.4	0.11	17.1	0.24	94	0.23
Some form or more	92 (13.2%)	17.2	0.02	17.7	0.64	89	0.97
Wealth PCA quintiles							
1	146 (21%)	16.1	Ref.	17.4	Ref.	89	Ref.
2	103 (14.8%)	16.3	0.76	17.5	0.80	88	0.90
3	151 (21.7%)	17.3	0.04	17.8	0.06	93	0.18
4	149 (21.4%)	17.0	0.13	17.8	0.05	92	0.45
5	146 (21.0%)	17.0	0.09	17.8	0.08	92	0.38
Employed for wages in past three months							
Yes	570 (82.1%)	16.5	Ref.	17.4	Ref.	92	Ref.
No	124 (17.9%)	18.0	< 0.001	18.8	< 0.001	88	0.07
Pregnancy intention							
Intends to get pregnant in next 12 months	82 (11.8%)	15.8	< 0.01	17.0	< 0.01	71	< 0.001
Does not intend to get pregnant in next 12 months	612 (88.2%)	16.9	Ref.	17.8	Ref.	94	Ref.
Happiness about pregnancy in next year							
Very happy	127 (18.3%)	16.8	Ref.	17.7	Ref.	80	Ref.
A little bit happy	47 (6.8%)	16.9	0.71	17.5	0.72	91	0.07
Not at all happy	521 (75.0%)	16.8	0.97	17.7	0.89	94	< 0.001
Timing for next child							
No more children	246 (35.5%)	16.7	Ref.	18.0	Ref.	94	Ref.
As soon as possible	31 (4.5%)	14.2	< 0.001	15.8	< 0.001	71	< 0.001
Defined time	271 (39.1%)	17.8	< 0.01	18.2	0.36	91	0.15
Undecided	145 (20.9%)	15.7	< 0.01	16.5	< 0.001	92	0.45
Method type							
None	61 (8.9%)	15.6	Ref.	17.1	Ref.	–	–
Permanent	126 (18.3%)	16.8	0.03	18.0	0.02	–	–
Long-acting	196 (28.5%)	17.0	< 0.01	17.6	0.12	–	–
Short-acting	306 (44.4%)	16.9	0.01	17.7	0.05	–	–

Higher numbers on the freedom from coercion and communication subscales represent higher levels of the autonomy in each respective subscales. P-values from bivariable regressions with standard errors clustered on village

**Fig. 1:**
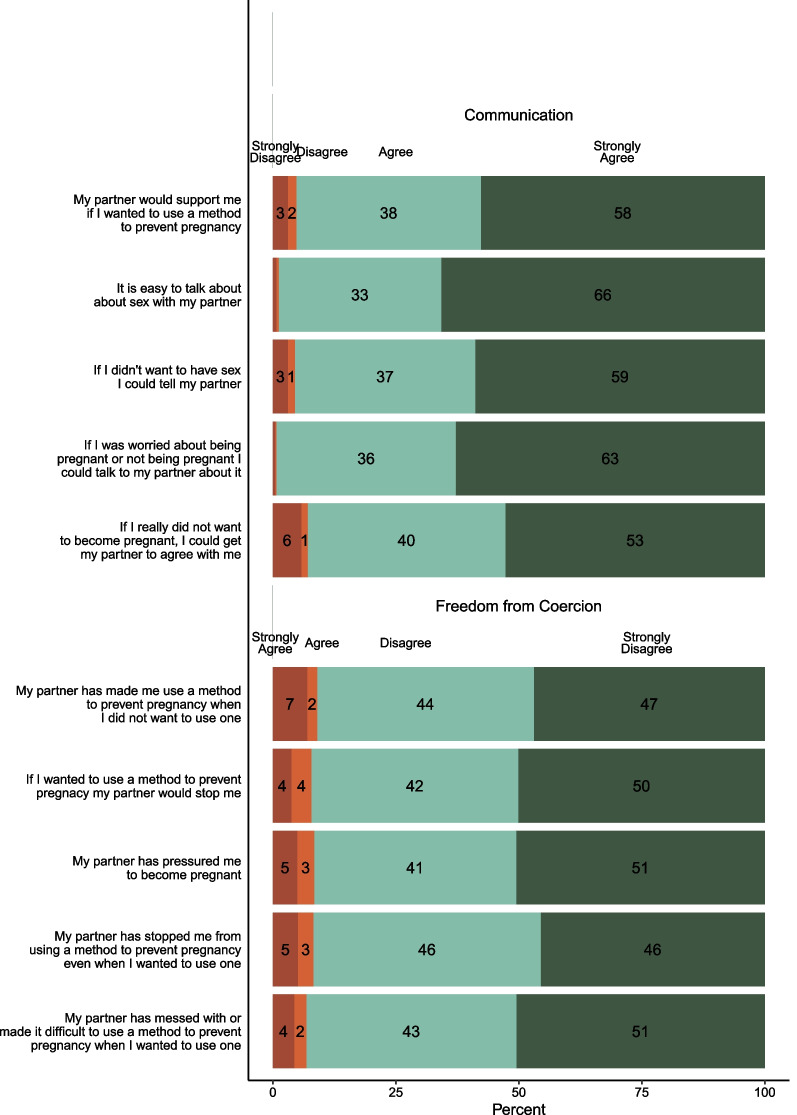
Reproductive Autonomy Scale items by subscale

The *freedom from coercion* subscale ranged from 5 to 20 (possible range: 5–20) had a mean of 16.8 and a median of 15.5. The *communication* subscale had a mean of 17.7, a median of 17.0, and ranged from 10 to 20 (possible range: 5–20). Distributions of the responses to the individual Reproductive Autonomy Scale items are included in Fig. 1.

Most participants disagreed or strongly disagreed with most items on the *freedom from coercion* subscale, indicating low levels of coercion. However, 9% of respondents agreed or strongly agreed that their partner made them use a method to prevent pregnancy when they did not want to use one and the same percentage agreed or strongly agreed that their partner had pressured them to become pregnant. Eight percent agreed or strongly agreed that if they wanted to use a method to prevent pregnancy, their partner would stop them.

Most participants strongly agreed or agreed with the items on the *communication* subscale, indicating high levels of communication. The most disagreement on the *communication* subscale was observed with the statement “If I really did not want to get pregnant, I could get my partner to agree with me” with 7% of participants disagreeing or strongly disagreeing with this statement.

### Construct validity of the Reproductive Autonomy Scale

Fit statistics from the models suggested that the two-factor correlated model was significantly better than the uncorrelated model (Table 2). Standardized item loadings for all items varied between 0.67 and 0.88, with the strongest loadings recorded for “If I was worried about being pregnant or not being pregnant, I could talk to my partner about it” (0.88) and “My partner has messed with or made it difficult to use a method to prevent pregnancy when I wanted to use one” (0.88). The two subscales had high levels of internal consistency. The Cronbach’s alpha for the *freedom from coercion* subscale and the *communication* subscale was 0.82 and 0.73.

**Table 2:** Confirmatory factor analysis fit statistics

	Items	Chi square	Df	CFI	TLI	RMSEA	SRMR
Model							
Two factor—correlated factors	10	208.1*	34	0.98	0.98	0.09	0.03
Two factor—oblique factors	10	5341.9*	35	0.41	0.25	0.47	0.36

Table displays fit statistics for two-factor models (communication and freedom from coercion). Degrees of Freedom (Df), confirmatory factor index (CFI), Tucker Lewis Index (TLI) and root mean square error of approximation (RMSEA), standardized root mean square residual (SRMR)

### Associations between the Reproductive Autonomy Scale and contraceptive use

In examining bivariate relationships between the two subscales and other variables, there were no significant differences by age and happiness related to a pregnancy in the next year (Table 1). Those who were in a non-cohabitating relationship or engaged (p < 0.01) and had no education (p = 0.02) had lower *freedom from coercion* scores. Among respondents who were employed for wages in the past three months, the average *freedom from coercion* score was 16.5 compared to 18.0 among those that were not employed for wages (p < 0.001). Women who intended to get pregnant in the next year had lower *freedom from coercion* scores than those that did not intend to get pregnant in the next year (p < 0.01).

The majority of women in the sample reported current use of contraception (91%) (Table 1). Those who were in a non-cohabitating relationship or engaged had lower rates of contraceptive use than those who were married (79% versus 92%; p < 0.05). Contraceptive use varied by pregnancy intention: 71% of respondents intending to get pregnant in the next 12 months reported currently using contraception, while 94% of those who did not intend to get pregnant in the next 12 months reported using contraception (p < 0.001). Eighty percent (80%) of respondents who reported they would be very happy if they got pregnant in the next year, regardless of their separately reported pregnancy intention, reported using contraception.

Among participants, 29% were using long-acting methods, 44% were using short-acting methods, and 18% were sterilized (Table 1). All long-acting method users reported using implants and most short-term users reported using injections (88%) (data not shown).

In unadjusted models, a higher score on the *freedom from coercion* subscale was associated with contraceptive use (OR = 1.15, 95% CI: 1.05–1.26), and results were largely unchanged when sociodemographic and partnership characteristics were included in the model (aOR = 1.16, 95% CI: 1.07–1.26) (Table 3). In other words, for every additional point on the *freedom from coercion* subscale, the odds of using contraception increased by 16% (95% CI: 7–26%). The interaction between the *freedom from coercion* subscale and pregnancy intention in the next year was not significant, thus the interaction was dropped from the model (results not shown). The pregnancy intention main effect was retained in the model. In the adjusted model, women who had higher scores on the freedom from coercion subscale were still more likely to use contraception, though the effect was attenuated (aOR 1.13, 95% CI: 1.03–1.23).

**Table 3:** Bivariable and multivariable models examining the association between reproductive autonomy and current contraceptive use among women in the UTHA cohort, n = 688

	Unadjusted model OR 95% CI	Adjusted for sociodemographic and partnership characteristics OR 95% CI	Adjusted for sociodemographic, partnership, and pregnancy intention OR 95% CI
Freedom from coercion models			
RA Freedom from Coercion Subscale	1.15** 1.05–1.26	1.16*** 1.07–1.26	1.13* 1.03–1.23
Number of living children	–	1.24 0.93–1.66	1.09 0.83–1.44
Employed in past 3 months for wages	–	0.45* 0.26–0.77	0.51* 0.28–0.92
Education	–	0.99 0.91–1.07	0.95 0.88–1.03
Wealth score	–	1.16 0.95–1.41	1.23 0.98–1.53
Relationship status			
Married with one partner		Ref.	Ref.
Married with more than one partner	–	0.90 0.39–2.08	0.81 0.36–1.29
In a relationship/engaged		0.43 0.14–1.31	0.40 0.13–0.63
Intends to get pregnant in next 12 months	–	–	0.21*** 0.11–0.39
Communication models			
RA Communication subscale	1.14* 1.02–1.28	1.16* 1.02–1.30	1.10 0.97–1.25
Number of living children	–	1.23 0.91–1.66	1.08 0.89–1.31
Employed in past 3 months for wages	–	0.44* 0.26–0.74	0.55 0.27–1.11
Education	–	1.00 0.91–1.09	0.95 0.86–1.05
Wealth score	–	1.15 0.96–1.39	1.23* 1.01–1.50
Relationship status			
Married with one partner		Ref.	Ref.
Married with more than one partner	–	0.93 0.42–2.04	0.83 0.39–1.77
In a relationship/engaged		0.46 0.16–1.30	0.43 0.17–1.10
Intends to get pregnant in next 12 months	–	–	0.17*** (0.09–0.33)

In the unadjusted model with the *communication* subscale, a higher score on the subscale was associated with contraceptive use (OR = 1.14, 95% CI: 1.02–1.28), and results were largely unchanged when sociodemographic and partnership characteristics were included in the model (Table 3). The interaction between pregnancy intention in the next year and the communication subscale was not statistically significant; however, when the pregnancy intention main effect was introduced in the model, the *communication* subscale was no longer significantly associated with contraceptive use.

In sensitivity analyses testing the use of alternative measures of pregnancy intention (i.e., pregnancy happiness), the results with the *freedom from coercion* and *communication* subscales were largely unchanged; however, the effect of the communication subscale remained statistically significant (results not shown). Results were unchanged for both the *communication* and *freedom from coercion* models when excluding women who were sterilized (results not shown).

## Discussion

Our results confirmed the construct validity of two subscales of the Reproductive Autonomy Scale—*communication* and *freedom from coercion—*in a sample of women in rural Malawi. Further, our results illuminate the ways in which—*freedom from coercion* and *communication*—are associated with contraceptive use in this population. Higher levels of *freedom from coercion* were associated with current contraceptive use—and these results were consistent when we adjusted for sociodemographic, partnership, and pregnancy intention variables.

Our findings that women who reported reproductive coercion were less likely to be using contraception illuminate the importance of the relationship between reproductive coercion and contraceptive non-use. Reproductive coercion is a deliberate action or an attempt to influence or control a person’s reproductive choices or interfere with their reproductive autonomy [14, 40, 41]. Reproductive coercion has been shown to be associated with higher odds of recent unintended pregnancy and lower odds of contraceptive use in India [42], a higher likelihood of use of female-controlled methods in Bangladesh, India, and Nepal [43], and covert use of contraceptive in Nigeria [44]. In Kenya, men’s desire to continue having children has been shown to contribute to reproductive coercion [45]. Similarly, findings from the UTHA study also indicate that women perceive men as barriers to contraceptive use, noting partner disapproval of contraception, partially because of men’s desire to continue childbearing [8]. Our findings suggest that interventions to address reproductive coercion, such as sensitizing health care providers about reproductive coercion, ensuring patients’ health care information is kept confidential, respecting women’s autonomy in making decisions about contraception, screening for reproductive coercion, considering the need to account for covert use in contraceptive counseling, and offering alternative mechanisms for women to store their health card or contraceptive supplies [46, 47], may be important strategies to facilitate access to contraception among women who do not want to become pregnant and who want to use contraception. Health education programs with male partners to facilitate positive partner communication and involvement may also help address forms of reproductive coercion [9]. Given the prevalence of reported intimate partner violence (IPV) in Malawi [48] and the co-occurrence IPV has with reproductive coercion in other settings [40, 49], future research should study these jointly to under the effect they have on contraceptive use and other measures of reproductive agency.

In this study, the *communication* subscale was significantly related to contraceptive use in some, but not all models, and the direction of the relationship was consistent. This suggests that being able to communicate with a partner about contraception and reproductive goals may be associated with contraceptive use. Given the theoretical and empirical findings in other studies that couple communication about contraception and reproductive behavior is associated with contraceptive use [9, 28], the association between communication about reproductive matters and sex and contraceptive behavior in this setting deserves further investigation. The questions in this scale are largely hypothetical and assume that women have desire to make reproductive goals. In reality, any autonomy women have may be completely constrained by partners, other family members, and social norms [50]. It may also be the case that other family members, including a mother-in-law, may play a large role in household decision-making which is not explored in this scale [51]. Additionally, in contexts where men play a substantial role in decisions around when to have children and, to some extent, the use of contraception, there may be nuances in how women exercise power in their relationship that is not captured in this scale. For instance, literature has documented how covert use of contraception may be a strategy for women to avoid conflict with their husbands [52, 53].

Pregnancy intention is also a complex construct, and its association with reproductive autonomy invites attention in future research. We did not find evidence that pregnancy intention moderated the relationship between the *communication* or *freedom from coercion* subscales and contraceptive use; however, it may be important to examine the relationships between these constructs using other statistical methods (e.g., structural equation modeling) that can assess if, for example, an underlying latent characteristic of empowerment jointly determines both reproductive autonomy and stronger or more resolute pregnancy intentions [26]. A number of items included on the *communication* subscale frame communication around pregnancy intentions assuming strong feelings of wanting to avoid a pregnancy. In a context like rural Malawi where fertility is associated with social status and stability of a marriage, these questions may not adequately capture how women consider pregnancy [30]. In fact, in this sample 70% of women who said they intended to get pregnant in the next year, and 71% who intended to get pregnant as soon as possible, reported current use of a contraceptive method. Social desirability bias may have impacted responses to pregnancy intention questions as respondents may have felt it was more acceptable to say they desired more children to the enumerator.

Participants in this study reported extremely high levels of contraceptive use. We note that some measurement error may exist in these reports as qualitative work with this population suggests that this may be because “current” contraceptive use is interpreted as use within the last few months regardless of whether the respondent is using it on the day of the survey [54]. Other studies, however, document high contraceptive use in this population. In the 2015–2016 Demographic and Health Survey, 75% of women who met similar inclusion criteria for this study (author’s calculations including those who were not currently pregnant, lived in the rural central region, ever had sex, were currently married or living with a partner, were not menopausal or sterilized, and who had at least one child) reported current use of a contraceptive method. Past waves of the UTHA cohort study indicate similarly high prevalence of use [22, 55]. In addition to the misclassification around ‘current use’, a reporting bias may be present whereby cohort participants, located within a hospital catchment area and frequently surveyed, were providing socially desirable responses regarding contraception use. Alternatively, participants may have altered their contraceptive behavior as a result of living in the study area (i.e., Hawthorne Effect) or because the health facility in the area provided a range of free contraceptive methods [56]. The small number of contraceptive non-users may have limited the power of this study to detect true differences between users and non-users.

Due to the cross-sectional nature of this study, we are limited in the conclusions we can draw about the temporality of the relationship between reproductive autonomy and contraceptive use. However, the *freedom from coercion* subscale is framed to capture past experiences, allowing some confidence in temporality to be established (i.e., coercion preceding contraceptive use). Also, assessing the performance of the Reproductive Autonomy Scale in this setting is limited by the choice we made based on local collaborator input and assessments of local relevance to omit the *decision-making* subscale. The exclusion of this subscale hinders comparisons to other settings. There may also be cross-cultural differences that influence how the scale was answered by survey respondents. It may be that responses to questions related to reproductive coercion on the *freedom from coercion* subscale are influenced by the social importance of childbearing and expectations within relationships in Malawi. Understanding reproductive coercion in this context may require additional qualitative work. Answers to these questions in particular, may also have been influenced by the survey modality. Respondents may not have felt comfortable sharing if they had experienced items on the *freedom from coercion* scale to an enumerator. Additionally, it is possible that women who are most vulnerable to abuse did not participate in the study, creating ceiling effects in our measurement. Finally, no measures of power differentials (e.g. age difference between spouses) or other dimensions of agency within sexual relationships (e.g. household decision-making power) were included in the survey. Future work that includes these measures could document the extent to which reproductive autonomy acts independently of other measures of agency within a sexual relationship to affect contraceptive use.

## Conclusion

We found that the Reproductive Autonomy Scale was valid among a sample of partnered women in rural Malawi and that higher levels of *freedom from coercion* and in some cases, higher levels of *communication,* were associated with contraceptive use. This points to the importance of examining reproductive autonomy in future work and ensuring that health care providers are aware and have tools to help patients enact their reproductive goals. Interventions to address reproductive coercion may be important strategies to facilitate access to contraception among women who do not want to become pregnant and who want to use contraception.

## Data Availability

More information about the data can be found at https://u.osu.edu/utha/. Please reach out to Alison Norris (Norris.570@osu.edu) for further information regarding data availability.

## Appendix

See Table 4.

**Table 4:** Reproductive autonomy items

Domain	Items
Freedom from coercion	• My partner has stopped me from using a method to prevent pregnancy when I wanted to use one • My partner has messed with or made it difficult to use a method to prevent pregnancy when I wanted to use one • My partner has made me use a method to prevent pregnancy when I did not want to use one • If I wanted to use a method to prevent pregnancy my partner would stop me • My partner has pressured me to become pregnant
Communication	• My partner would support me if I wanted to use a method to prevent pregnancy • It is easy to talk about sex with my partner • If I didn’t want to have sex I could tell me partner • If I was worried about being pregnant or not being pregnant I could talk to my partner about it • If I really did not want to become pregnant I could get my partner to agree with me
